# Molecular Basis for Viral Selective Replication in Cancer Cells: Activation of CDK2 by Adenovirus-Induced Cyclin E

**DOI:** 10.1371/journal.pone.0057340

**Published:** 2013-02-20

**Authors:** Pei-Hsin Cheng, Xiao-Mei Rao, Kelly M. McMasters, Heshan Sam Zhou

**Affiliations:** 1 Department of Pharmacology and Toxicology, University of Louisville School of Medicine, Louisville, Kentucky, United States of America; 2 Department of Surgery, University of Louisville School of Medicine, Louisville, Kentucky, United States of America; 3 James Graham Brown Cancer Center, University of Louisville School of Medicine, Louisville, Kentucky, United States of America; 4 Department of Microbiology and Immunology, University of Louisville School of Medicine, Louisville, Kentucky, United States of America; Wuhan Bioengineering Institute, China

## Abstract

Adenoviruses (Ads) with deletion of *E1b55K* preferentially replicate in cancer cells and have been used in cancer therapies. We have previously shown that Ad E1B55K protein is involved in induction of cyclin E for Ad replication, but this E1B55K function is not required in cancer cells in which deregulation of cyclin E is frequently observed. In this study, we investigated the interaction of cyclin E and CDK2 in Ad-infected cells. Ad infection significantly increased the large form of cyclin E (cyclin EL), promoted cyclin E/CDK2 complex formation and increased CDK2 phosphorylation at the T160 site. Activated CDK2 caused pRb phosphorylation at the S612 site. Repression of CDK2 activity with the chemical inhibitor roscovitine or with specific small interfering RNAs significantly decreased pRb phosphorylation, with concomitant repression of viral replication. Our results suggest that Ad-induced cyclin E activates CDK2 that targets the transcriptional repressor pRb to generate a cellular environment for viral productive replication. This study reveals a new molecular basis for oncolytic replication of *E1b*-deleted Ads and will aid in the development of new strategies for Ad oncolytic virotherapies.

## Introduction

Human adenoviruses (Ads) are double-stranded linear DNA viruses that are able to infect and replicate in a wide variety of cell types *in vitro* and *in vivo*, including post-mitotic cells. After infection, viral early proteins interact with cellular factors to create favorable environments for viral replication [1]. The Ad *E1* region contains two sets of genes, *E1a* and *E1b*, that are dedicated to cell cycle control, apoptotic inhibition, and cellular and viral gene regulation [2]. Ads with *E1* modifications that preferentially replicate in cancer cells have been used for cancer gene therapy.

The viral *E1a* gene is expressed immediately after infection. The primary role of *E1a* gene products is to regulate expression of multiple cellular and viral genes [1]. Instead of directly binding to specific DNA sequences in transcriptional regulation elements, E1A proteins interact with several key regulators of cell proliferation [3], [4]. The well-known cellular factors to which E1A proteins bind are products of the retinoblastoma (*Rb*) gene and its structurally related genes, *p107* and *p130* [5], [6]. By sequestering the retinoblastoma protein (pRb), E1A activates transcriptional regulator E2F proteins. Studies have suggested that the pRb/E2F complex actively represses transcription from target genes and mediates G_1_ arrest triggered by p19 (ARF), p53, p16INK4a, TGF beta, or cell contact [7]–[9]. Recently Pelka *et al.* (2011) indicated that E1A can directly bind to E2F/DP complexes by interacting with DP-1, resulting in the activation of E2F-responsive gene expression independently of binding to pRb [10]. Several groups have shown that expression of *E1a* gene triggers the accumulation of p53 protein and p53-dependent apoptosis [11], [12] either by activating p53 transcription or preventing p53 from being degraded by the proteasome [11]–[14].

Ad E1B55K has been shown in some studies to counteract the E1A-induced stabilization of p53 [11], [15]. E1B55K protein may inhibit the functions of p53 through at least three distinct mechanisms. E1B55K reportedly binds the amino terminus of p53 [16], and this binding may repress p53 transcriptional activation, as suggested in transcription assays [17] and transient transfection studies [18]. E1B55K may also interfere with p53 function by cooperating with viral E4orf6 protein to cause proteolytic degradation of p53 protein [19]–[21]. A recent study has showed that E1B55K alone functions as an E3 SUMO1-p53 ligase that interacts with promyelocytic leukemia nuclear bodies to inactivate p53 and stimulate its nuclear export [22]. Thereby, E1B55K blocks the expression of p53-regulated genes and, consequently, counteracts the p53-dependent apoptosis induced by E1A, allowing efficient viral replication [16], [17].

Ad *dl*1520 (ONYX-015) contains an 827-bp deletion and a point mutation generating a premature stop codon in the E1B55K coding sequence, preventing expression from the gene [23]. It was originally proposed that the *E1b55K*-deleted Ads could replicate only in p53-deficient tumor cells, as the E1B55K-mediated degradation of p53 protein was not required in those cancer cells [24], [25]. *E1b55K*-deleted oncolytic Ads have been tested in human clinical trials and are being marketed for cancer treatment in China after approved by China's State Food and Drug Administration (SFDA) [26]. However, the original hypothesis was challenged by several studies showing that *E1b55K*-deleted Ads are able to replicate in cells regardless of their p53 status [27]–[30]. Further studies have shown that the accumulation of p53 protein, after infection with Ads carrying mutated *E1b55K* genes that are unable to repress p53, can neither efficiently induce apoptosis nor transcriptionally activate expression of p53-responsive genes in Ad-infected cells [31], [32]. Thus, these results suggest that blocking of p53 activity by E1B55K protein is unlikely to be the major requirement for viral replication. The mechanism(s) of *E1b55K*-deleted viral replication in cancer cells is still not established, even though the vectors have already been applied in the clinic for human cancer treatment [26].

Previously, we have shown that Ad E1B55K is involved in the induction of cell cycle-related genes, including cyclin E and CDC25A [33]. Ad E1B55K mediates the large form of cyclin E protein (cyclin EL) induction in Ad-infected cells [34]. Cyclin E and the large form cyclin EL are generated from the alternative splicing. The translation of cyclin EL is initiated at an ATG codon located in exon 2 and cyclin E is from the ATG codon in exon 3 [35]. The E1B55K function is required for cyclin EL induction in normal cells, but is not required in cancer cells with deregulated cyclin E. Failing to efficiently induce cyclin EL expression in the normal cells, replication of *E1b55K*-deleted oncolytic Ads is restricted. However, *E1b55K*-deleted oncolytic Ads can efficiently induce cyclin EL in cancer cells and carry out sufficient oncolytic replication. We proposed that cyclin E deregulation in cancer cells may be an important molecular basis for the selective oncolytic replication of *E1b55K*-deleted Ads [34].

Cyclin E regulates cell cycle progression, DNA replication [36], [37], and centrosome duplication [38], [39]. Expression of cyclin E is strictly controlled in normal cells. The level of cyclin E rises at late G_1_ phase, peaks at the G_1_/S phase to promote the S-phase entry, and decreases thereafter [35], [40]. Deregulation of cyclin E is frequently detected in many types of cancers, as cyclin E gene amplification [41], overexpression of cyclin E mRNA or protein levels [42], [43], decrease of cyclin E turnover [44], and the presence of more active forms of cyclin E [45]–[47]. Constitutive overexpression of cyclin E was shown to induce chromosome instability and impair normal cell cycle progression [48], [49]. The hypothesis that abnormal cyclin E expression can trigger tumors has also been supported by transgenic animal studies [50]–[52].

One function of cyclin E is to bind and activate cyclin-dependent kinase 2 (CDK2) [53]. The cyclin E/CDK2 complex then phosphorylates substrates such as pRb and leads to transcriptional activation of downstream genes. Studies also indicate that cyclin E has CDK2-independent functions [54], [55]. *In vivo* animal studies indicate variance between the phenotypes of cyclin E null (cyclin E1^−/−^ E2^−/−^) mice and CDK2 null (CDK2^−/−^) mice. Mice lacking CDK2 are viable, with normal development except defective germ cell development [56], [57]; yet knockout of cyclin E1 and E2 genes in mice causes embryonic lethality owing to the deficiency in endoreplication of trophoblast giant cells and megakaryocytes [58]. Matsumoto *et al.* (2004) identified a centrosomal localization signal (CLS) domain in cyclin E [59]. This CLS domain allows cyclin E to target the centrosome and promote S phase entry in a CDK2-independent manner. Additionally, Geng *et al.* (2007) showed that a cyclin E kinase-deficient mutant (KD-E) is able to partially restore minichromosome maintenance protein (MCM) loading and S phase entry in cyclin E null cells [54]. Thus, cyclin E has CDK2-dependent and independent functions in S phase entry and DNA replication. An important question is whether Ad-induced cyclin E may activate CDK2 and whether the cyclin E-CDK2 interaction may play a crucial role in Ad replication. This question is especially important in the development of oncolytic virotherapy strategies.

We report here that Ad-induced cyclin E binds with and activates CDK2 that targets transcription repressor pRb, which in turn can regulate expression of cellular and viral genes. The results suggest that the interaction between the Ad-induced cyclin E and CDK2 is to generate a suitable environment for Ad productive replication.

## Materials and Methods

### Cell lines and culture conditions

HEK 293 (ATCC no. CRL-1573), human lung fibroblast WI-38 (ATCC no. CCL-75), and human lung cancer A549 (ATCC no. CCL-185) cell lines were purchased from the American Type Culture Collection (Rockville, MD). WI-38 cells were cultured in minimal essential medium (MEM) Alpha GlutaMAX with 0.1 mM non-essential amino acids and 1.0 mM sodium pyruvate. HEK 293 and A549 cells were cultured in minimal essential medium Alpha. All media were supplemented with 10% fetal bovine serum (FBS) and penicillin/streptomycin (100 U/ml). Cells were cultured in a 5% CO_2_ incubator at 37 °C. All cell culture reagents were obtained from Gibco BRL (Bethesda, MD).

### Adenoviral vectors

Wild-type adenovirus type 5 (Adwt, ATCC no. VR-5) was used as a replication-competent control. AdCMV/GFP, an Ad vector with E1 deletion carrying a green fluorescent protein (GFP), was used as a replication-defective control. Adhz63, an oncolytic Ad vector with the deletion of *E1b55K* region, was constructed by our laboratory [60].

### Viral infection and titration

Cells were seeded into 6-well plates at a density of 2.5×10^5^ (cells/well) and cultured under the indicated conditions. Subsequently, cells were mock-infected or infected with AdGFP, Adwt, or Adhz63 at a multiplicity of infection (MOI) of 5. Cytopathic effect (CPE) was observed at designed time points and photographed with an inverted microscope (Olympus CKX41). Total infected cells and culture supernatants were collected at 48 h postinfection (p.i.) and lysed to release virus particles with three cycles of freezing and thawing. The viral titers were determined by the infective unit method as described previously [61], [62]. Briefly, HEK 293 cells were seeded in 96-well plates at a density of 10^3^ (cells/well) and then infected with 5-fold serially diluted viruses. CPE was recorded and scored after incubation for 7 days. The reduction percentage in virus titer is calculated by the formula, reduction %  =  [(titer of control group – titer of experimental group)/titer of control group]×100%.

### Viral DNA synthesis assay

After viral infection, cells were collected at different time points. The viral DNA synthesis was determined with Southern blot; 1 µg of isolated genomic DNA was digested with the restriction enzyme *Pst*I and analyzed with 1% agarose gel, which was subsequently transblotted to a Hybond-N+ membrane (YA3609; Amersham Pharmacia Biotech, Arlington Heights, IL). The probe was prepared by digesting 0.5 µg pBHGE3 [63] with *Pst*I and labeled by following the protocol of Amersham AlkPhos Direct Labeling and Detection Systems (RPN 3690; GE Healthcare, Piscataway, NJ). The blot was prehybridized for 3 hrs at 63 °C. The hybridization and stringency washes were performed at 55 °C and followed by the chemiluminescent detection according to the manufacturer's protocol.

### Western blot analysis

Infected cells were harvested at indicated time points and lysed with CDK2 lysis buffer (20 mM Tris pH 7.5, 150 mM NaCl, 5 mM MgCl_2_, 0.5% Nonidet P-40, 0.1% Brij 35, 5 mM sodium glycerophosphate, 1 mM sodium vanadate, 1 mM dithiothreitol). The Western blot analyses were performed as described previously [64]. Briefly, 80 µg of cell lysates were electrophoresed through 12% SDS-polyacrylamide gels and transferred onto an Immobilon-P Membrane (Millipore, Billerica, MA). The primary antibodies used in this study were rabbit anti-cyclin E (M-20), CDK4 (C-22), mouse anti-cyclin D1 (DCS-6), PCNA (PC10), p21 (F-5), pRb (IF8) (Santa Cruz Biotechnology, Santa Cruz, CA), mouse anti-CDK2, p27 (BD Biosciences, San Jose, CA), pCDK2 T160 (Cell signaling, Danvers, MA), rabbit anti-phosphorylated pRb (phospho-pRb) S612, and actin (Sigma, St. Louis, MO), anti-phospho-pRb S795 (New England Biolabs, Beverly, MA), and anti-phospho-pRb T821 (Invitrogen, Carlsbad, CA). Actin was used as an internal control. The membranes were then incubated with anti-mouse immunoglobulin G (IgG) or anti-rabbit IgG peroxidase-linked species-specific whole antibody (GE Healthcare, Piscataway, NJ). Chemiluminescent detection was performed with ECL reagents according to the supplier's recommendations (GE Healthcare). The scanned band intensity was quantitated by Gel-pro Analyzer 4.0 software (Media Cybernetics, Bethesda, MD) according to the manufacturer's tutorial. Densitometric value for each band was expressed as integrated optical density (I.O.D.) and the results were normalized with actin. The final values represent the means of relative percentage change, from at least three independent experiments, compared with the mock group ± S.D.. Statistical difference was assessed with Student's t-test. A p-value of <0.05 was considered significant.

### Immunoprecipitation

A549 cells were seeded in 150 mm dishes at a cell density of 5×10^6^ (cells/dish) and then mock-infected or infected with AdGFP, Adwt, or Adhz63 at an MOI of 5. At 48 h p.i., cells were collected and lysed with CDK2 lysis buffer according to the method described in previous publications [34], [65]. Cell lysates (500 µg) were immunoprecipitated with cyclin E (HE111), the mouse monoclonal antibody (Santa Cruz), or anti-CDK2 antibody (BD Transduction Laboratories) at 4 °C for 4 h, followed by adding protein A Sepharose CL-4B (82506; Sigma) and incubating overnight. Immunocomplexes were analyzed by Western blot with anti-cyclin E and CDK2 antibodies.

### Small interfering RNA (siRNA) transfection

The siRNA oligonucleotides were synthesized by Eurogentec (Fremont, CA). Three different siRNA duplexes were designed to target CDK2 on nucleotides 399 to 419 (#1), 619 to 639 (#2), and 691 to 711 (#3) according to Genbank accession NM001798.2 (National Center for Biotechnology Information GenBank). A negative control siRNA duplex containing two strands of 19 complementary RNA bases with 3'dTdT overhangs was obtained from Eurogentec (SR-CL000-005). Cells were seeded into a 6-well plate at a density of 10^5^ (cells/well) and then transfected with 200 nM CDK2 siRNA duplexes or a non-specific control siRNA duplex with Lipofectamine 2000 (Invitrogen, Carlsbad, CA) according to the manufacturer's protocol. Cells were harvested at 48 h after transfection. Eighty µg of cell lysates were analyzed by Western blot with CDK2, pCDK2 T160, pRb, phosphorylated pRb (p-pRb), cyclin E, capsid proteins, and actin antibodies.

All above experiments, except specifically indicated, were repeated at least three times.

## Results

### Cyclin E/CDK2 complex formed in cells infected with adenoviruses

**Figure 1:**
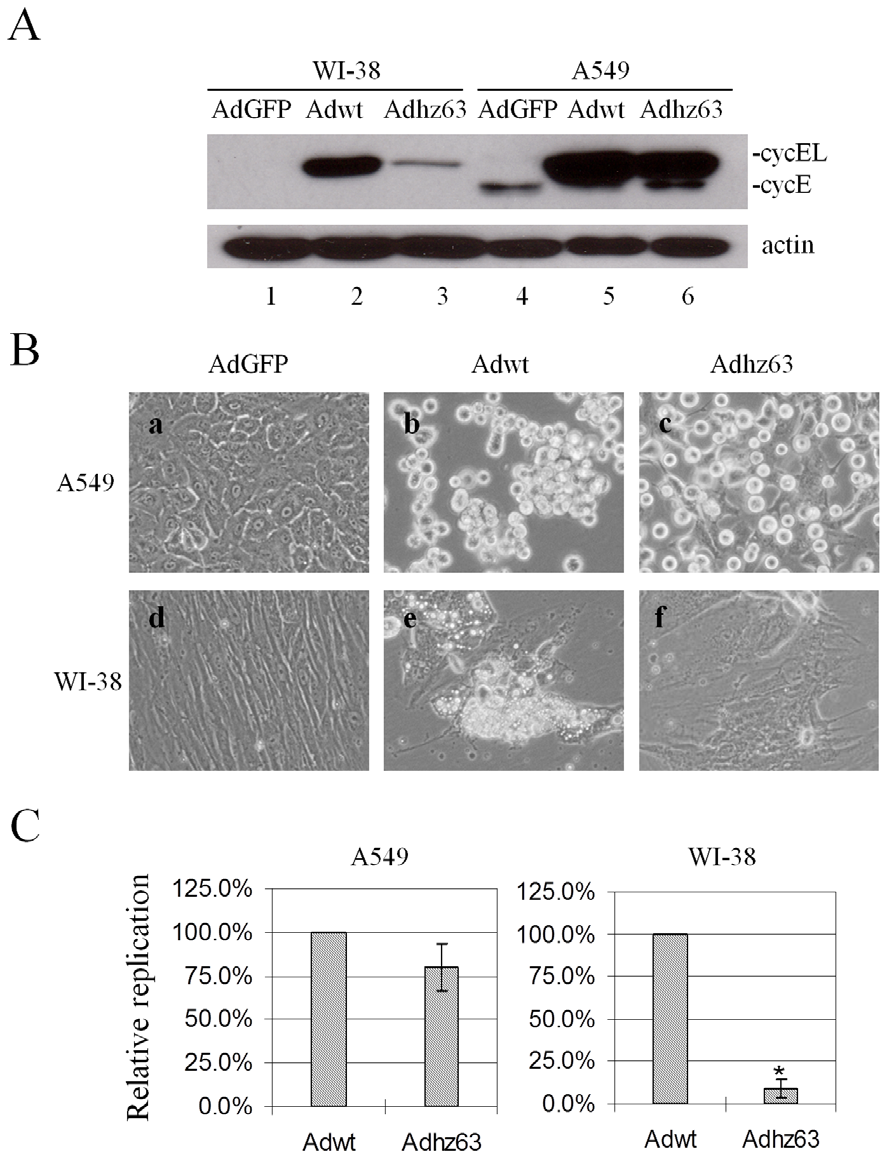
Virus replication is correlated with cyclin E overexpression. WI-38 or A549 cells were infected with AdGFP, Adwt or Adhz63 at an MOI of 5. (A) Cells were collected at 48 h and then analyzed by Western blot. Cell lysates were immunoblotted for cyclin E and actin. Actin was used as a loading control. (B) CPE was photographed at 72 h post infection (p.i.). All microscopy was originally at a magnification of x100. (C) Viral titers were determined at 72 h p.i. with the infection unit method. The value indicates the mean of three independent experiments, shown as the mean change percentage relative to Adwt control group ± S.D. * P<0.05 compared with the Adwt group, Student's *t*-test.

We have previously established the link between cyclin E and replication of adenoviruses [33], [34]. The data shown in Figure 1 recapitulate that Ad E1B55K participates in the induction of the large form of cyclin E protein (cyclin EL), which contributes to the efficient viral replication. Cyclin E and cyclin EL are generated from alternative splicing with different start ATG codons in exons 2 and 3 [35]. The N terminus of virus-induced cyclin EL is 15 amino acids longer than that of cyclin E protein. Cyclin E protein is constitutively expressed in A549 human lung cancer cells [34]. Wild-type Ad5 (Adwt), with the intact *E1b55K* region, induced significant cyclin EL expression in both of WI-38 human lung fibroblast cells and A549 human lung cancer cells (Fig. 1A, lanes 2 and 5), and caused efficient cytopathic effect (CPE) (Fig. 1B, panels b and e). Ad vector Adhz63 with *E1b55K*-deletion [60] also induced significant cyclin EL in A549 cells (Fig. 1A, lane 6) and caused efficient CPE of the cells (Fig. 1B, panel c). However, the vector failed to induce cyclin EL overexpression in WI-38 cells (Fig. 1A, lane 3) and their CPE (Fig. 1B, panel f). To compare the replication of Adwt and Adhz63 in A549 and WI-38 cells, the titers of these two viruses were determined. The Adhz63 replication was strongly repressed in WI-38 cells, showing only 10% of relative replication in comparison with Adwt (Fig. 1C). When we compared Adhz63 replication with that of Adwt in A549 cells, consistent with the CPE results, 80% of relative replication of Adhz63 was observed. Thus, Adhz63 replication is more repressed in WI-38 cells than in A549 cells. The result is consistent with our previous observation that cyclin EL induction in cancer cells is connected with the selective replication of *E1b55K*-deleted Ads in cancer cells [34].

**Figure 2:**
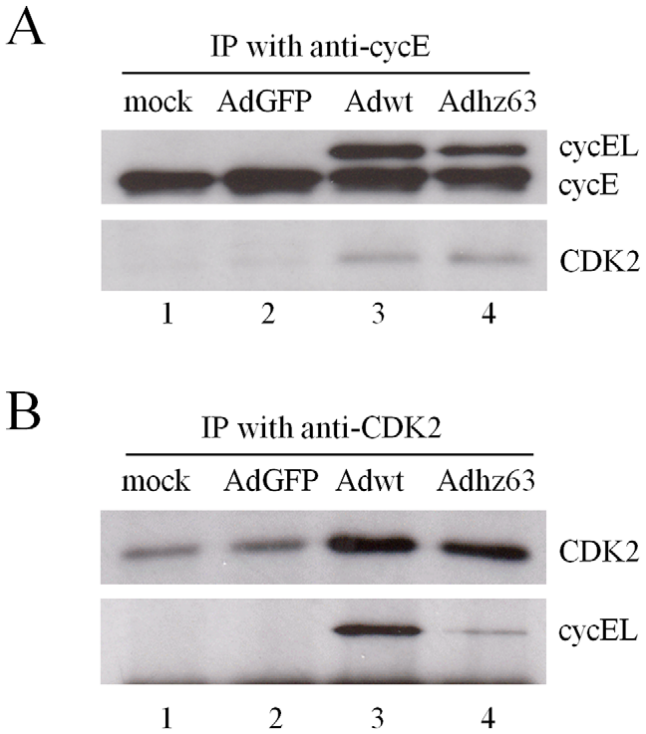
Cyclin E/CDK2 complex induced by viral infection in A549 cells. (A) A549 cell lysates were immunoprecipitated with anti-cyclin E antibody (1:50 dilution). Immunocomplexes were analyzed by Western blot with cyclin E and CDK2 antibodies. (B) The cell lysates were immunoprecipitated with anti-CDK2 antibody and immunoblotted for CDK2, cyclin E and cyclin EL.

Cyclin E can promote the S phase entry and participate in DNA replication via CDK2-dependent [53] and CDK2-independent pathways [59]. To study whether cyclin E function in Ad replication is CDK2 dependent or independent, we first sought to investigate the physical contact between CDK2 and cyclin EL in cells affected by Ads. Lung cancer A549 cells were mock-infected or infected with AdGFP, Adwt, or Adhz63. To understand how *E1b*-deleted Ads selectively replicate in cancer cells, we focused on A549 lung cancer cells in which both Adwt and the *E1b*-deleted Adhz63 can efficiently induce cyclin EL and replicate. At 48 h post-infection (p.i.), cells were collected and lysed. We first used anti-cyclin E antibody to immunoprecipitate cyclin E protein and analyzed the immunocomplexes with Western blot. The data show that cyclin E protein precipitated from cells mock-treated or treated with replication-defective AdGFP (negative controls) did not exhibit significant association with CDK2 protein (Fig. 2A, lanes 1 and 2). However, immunocomplexes from Adwt- and Adhz63-infected A549 cells contained both cyclin E and cyclin EL with an increase of CDK2 binding (Fig. 2A, lanes 3 and 4). To verify this cyclin E/CDK2 bonding, we also used anti-CDK2 antibody to pull down the protein complex and then examined the level of cyclin E proteins in the cyclin E-CDK2 complex. The immunoprecipitated CDK2 protein was increased in Adwt and Adhz63-infected cells with a concomitant precipitation of cyclin EL (Fig. 2B, lanes 3 and 4), especially for Adwt-infected cells (lane 3). The results show that replication-competent Adwt and Adhz63 induce cyclin EL expression and increase the formation of cyclin EL/CDK2 complex in A549 cancer cells, indicating that the cyclin EL induced in Ad-infected cells strongly associates with CDK2.

### Adenovirus-induced cyclin EL increases CDK2 phosphorylation

**Figure 3:**
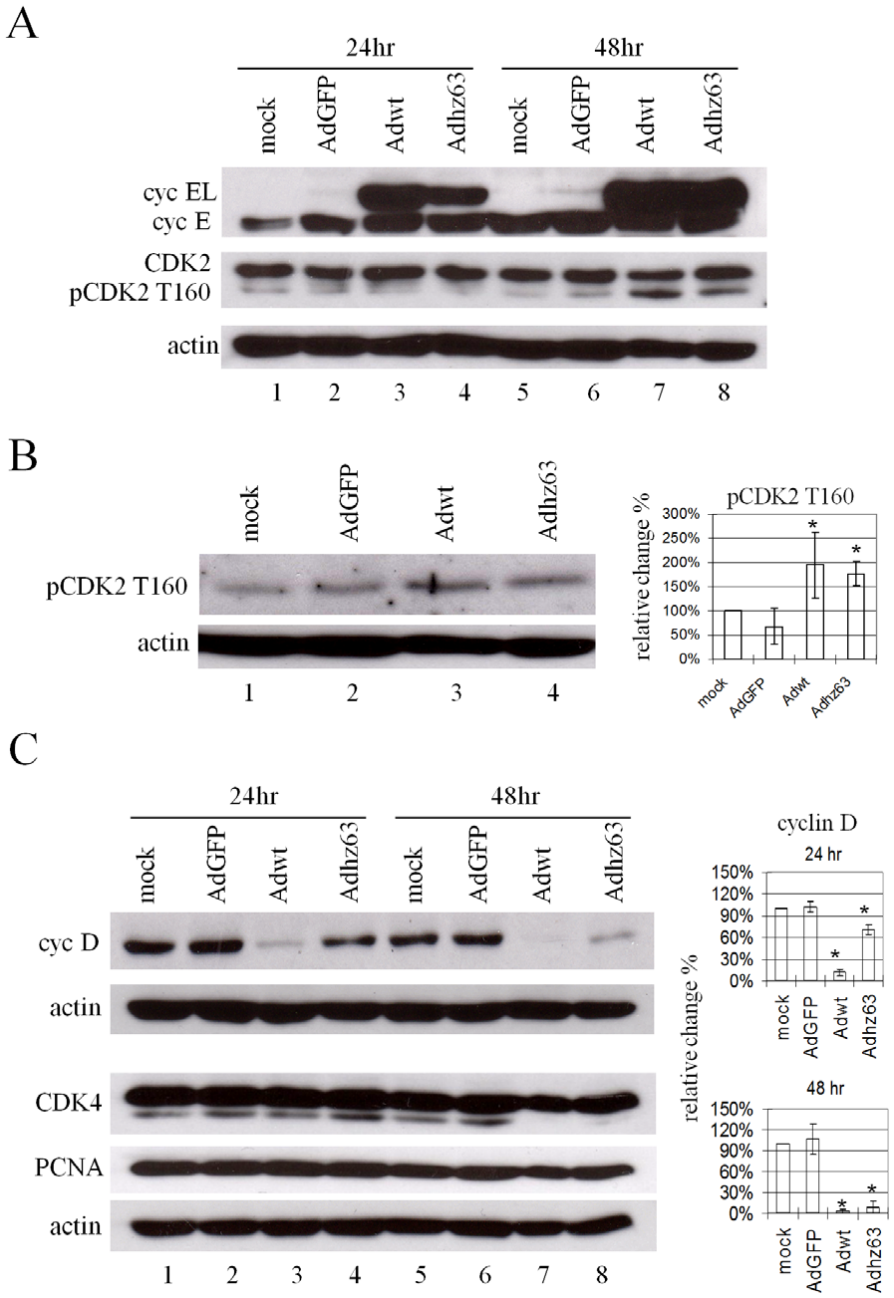
Effects of viral replication on cellular proteins related to G_1_/S phase. A549 cells were mock infected or infected with AdGFP, Adwt, or Adhz63 at an MOI of 5. Cells were collected at 24 h or 48 h p.i. and then analyzed by Western blot. Cell lysates were immunoblotted for (A) cyclin E and CDK2; (B) pCDK2 T160; and (C) cyclin D, CDK4 and PCNA. Actin was used as a loading control. The scanned band intensity was quantitated and the values represent the means of the relative change percentages compared with the mock group ± S.D. from three independent triplicate experiments. * P<0.05 compared with the mock-control group, Student's t-test.

CDK2 is activated by the phosphorylation at the T160 site and this phosphorylation increases its electrophoretic mobility, resulting in faster-migrating bands [66]. We investigated whether cyclin EL induction and the increased interaction between cyclin EL and CDK2 in A549 cells after Adwt and Adhz63 infection may promote CDK2 phosphorylation at the specific T160 site. Analysis of the cell lysates with Western blot demonstrated that the cyclin EL induction led to an increase of the faster-migrating CDK2, consistent with phosphorylated-CDK2 protein (pCDK2) T160 (the active form of CDK2), especially at 48 h p.i. (Fig. 3A, lanes 7 and 8). We verified the faster-migrating form of CDK2 with phospho-CDK2 (T160) antibody (#2561, Cell signaling). Densitometric analysis of these bands demonstrated that Adwt infection caused a 1.3 to 2.7-fold increase (P = 0.03) in the level of pCDK2 T160 and Adhz63 infection caused a 1.5 to 1.9-fold increase (P = 0.0026) compared with the mock-control group at 48 h p.i. (Fig. 3B, lanes 3 and 4). The result in figure 3B is consistent with that in figure 3A (lanes 7 and 8).

In addition to cyclin E, cyclin D is also involved in the transition of the G_1_-S phase. Thus, we also examined the level of cyclin D. Interestingly, the level of cyclin D was decreased after viral infection. Densitometric analysis of the bands demonstrated that Adwt and Adhz63 infection at 24 h decreased cyclin D protein to the levels of 11% (P = 0.0000025) and 71% (P = 0.001) of the mock infection, respectively (Fig. 3C, lanes 3 and 4). The levels of cyclin D in cells infected with Adwt or Adhz63 were further decreased at 48 h to 3% (P = 0.00000043) and 8% (P = 0.00002), respectively (Fig. 3C, lanes 7 and 8). Meanwhile, the level of CDK4 and the proliferating cell nuclear antigen (PCNA) did not significantly change in any of the groups. CDK4 is regulated and activated by cyclin D to process the G_1_-S transition [67], [68]; PCNA, known to regulate DNA replication and DNA repair, is associated with multiple cyclin/CDK complexes in the cell-cycle progression [69], [70]. The results show that Ads decreased cyclin D production and did not affect the levels of CDK4. Thus, Ad infection specifically induced cyclin EL that activated CDK2 via phosphorylation at its T160 site, suggesting a critical role of cyclin EL and CDK2 in Ad replication.

### Adenoviruses increase pRb phosphorylation

**Figure 4:**
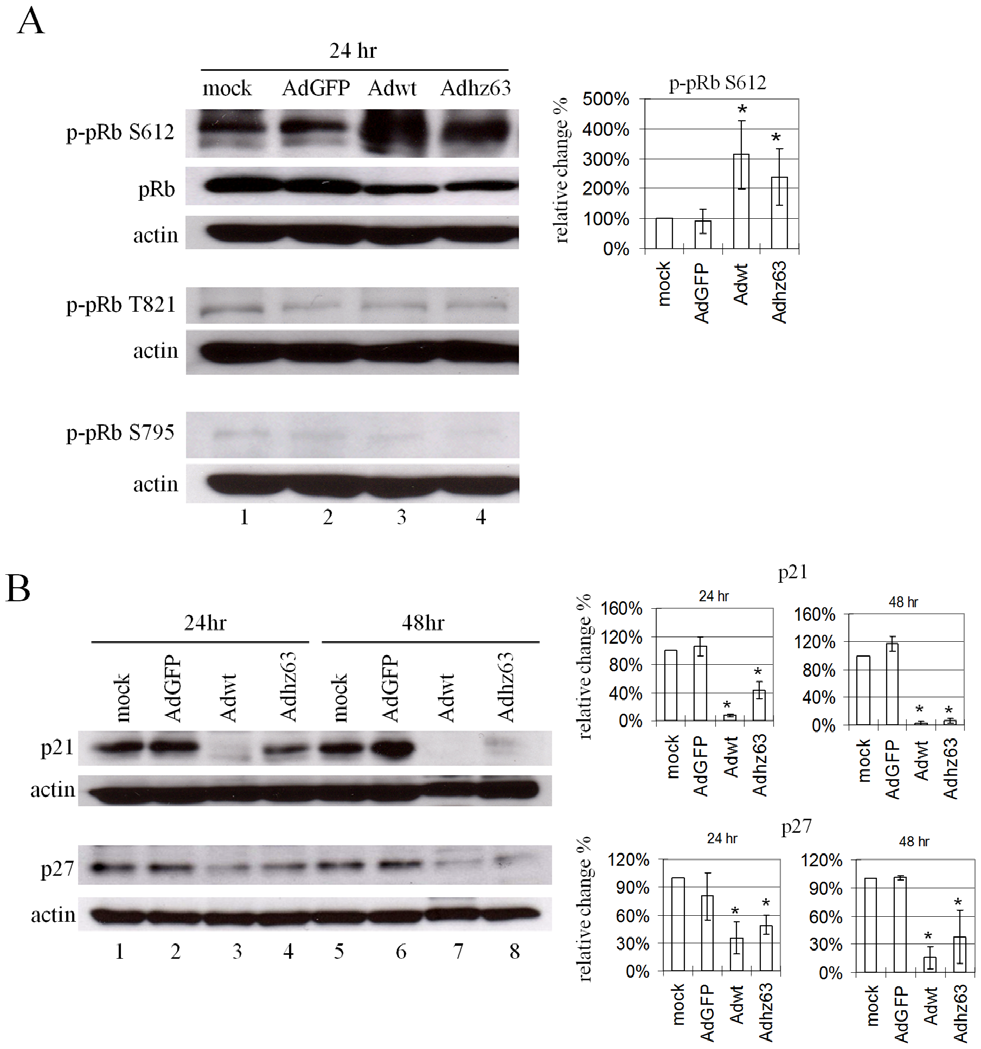
Effects of viral replication on pRb and CDK inhibitors. A549 cells were mock-infected or infected with AdGFP, Adwt, or Adhz63 and collected at 24 h or 48 h p.i., followed by Western blot analysis. Cell lysates were immunoblotted for (A) pRb, phospho-pRb (p-pRb) at S612, T821 and S795 or (B) p21 and p27. Actin was used as a loading control. * P<0.05 compared with the mock-control group, Student's t-test.

Cyclin E-activated phosphorylated CDK2 (pCDK2) is known to control the G_1_-S transition by phosphorylation of the downstream substrates. Considering that pRb is one of the well-known targets for pCDK2, we examined whether the increase of activated pCDK2 alters the phosphorylation of pRb at S612, which is a CDK2-preferred phosphorylation residue [71], [72]. We found that the level of phospho-pRb S612 was increased to 314% (P = 0.016) and 240% (P = 0.03) in cells infected with Adwt and Adhz63, respectively, even though the protein level of unphosphorylated pRb is decreased slightly (Fig. 4A, lanes 3 and 4). We could not detect any significant changes of p-pRb T821, another CDK2-preferred phosphorylation residue [71], [73], and the CDK4-preferred p-pRb S795 [74] (Fig. 4A). The results suggest the selection of the S612 site in pRb by Ad-activated CDK2 for protein phosphorylation.

### Adenoviruses repress CDK inhibitors

We also observed that the protein levels of both p21 and p27 are decreased in A549 cells infected with Adwt and Adhz63, especially for p21 (Fig 4B). p21 and p27 are the well-known CDK inhibitors, which negatively regulate the activity of cyclin E/CDK2 complexes to prevent the cell-cycle progression [75]. Densitometric analysis of these bands demonstrated that the level of p21 protein decreased to the levels of 8% (P = 0.0000002) and 44% (P = 0.00066) of the mock control in A549 cells after infection with Adwt and Adhz63 at 24 h, respectively (Fig. 4B, lanes 3 and 4). The p21 level was further repressed in A549 cells at 48 h after infection with Adwt (2%, P = 0.00000034) and Adhz63 (6%, P = 0.0000007) (Fig. 4B, lanes 7 and 8). Ad infection also decreased p27 protein levels to 36% (Adwt, P = 0.0015) and 49% (Adhz63, P = 0.00043) at 24 h; 16% (Adwt, P = 0.00012) and 37% (Adhz63, P = 0.0092) at 48 h in A549 cells (Fig. 4B). The results suggested that Ads activate the CDK2 by inducing cyclin EL and repressing p21 and p27.

### Interruption of cyclin EL and CDK2 interaction reduces adenoviral replication

**Figure 5:**
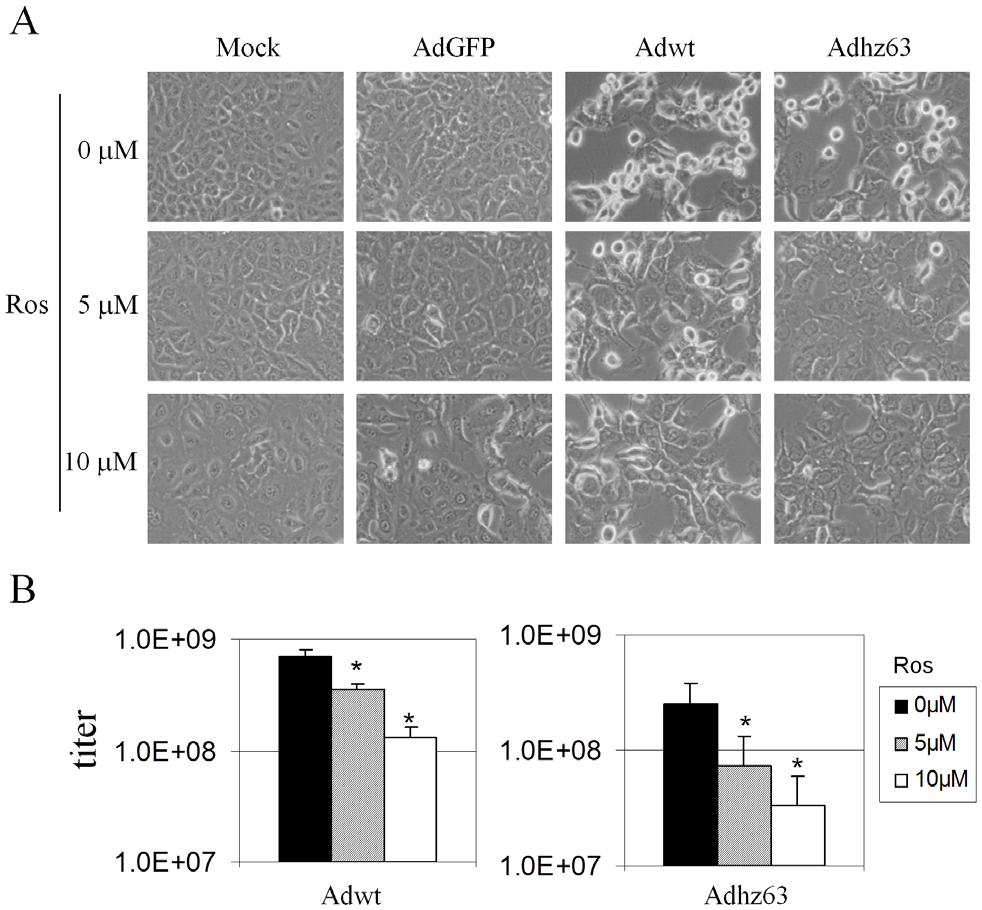
Effects of roscovitine on CPE and viral production. (A) Cells were treated with 0 µM (vehicle-control group treated with DMSO), 5 µM, or 10 µM roscovitine, and mock-infected or infected with AdGFP, Adwt or Adhz63 at an MOI of 5. All microscopy is originally at a magnification of x100 taken at 48 h p.i. (B) Viral titers were determined at 48 h p.i. with the infection unit method. The values represent the means ± S.D. of independent quadruplicate. * P<0.05 compared with the 0 µM roscovitine, Student's *t*-test.

To further investigate the role of CDK2 in viral replication, we used the CDK2 chemical inhibitor roscovitine (Ros; CYC202) to interrupt cyclin EL and CDK2 interaction. Ros is a purine derivative that inhibits the activity of CDK2 by binding to its active site [76]. Ros reduces phosphorylation on CDK2 [77] and blocks the androstenedione-induced increase of active phosphorylated CDK2 [78]. If activation of CDK2 is required for viral replication, blocking CDK2 activity should reduce it. Figure 5A, representing one of the four repeated experiments, shows that with increased Ros, CPE caused by Adwt and Adhz63 infection was partially inhibited. Figure 5B shows that treatment with 5 µM of Ros led to a 50% reduction in Adwt titer (P = 0.0002) and 71% reduction in Adhz63 titer (P = 0.034) when compared with the vehicle-control group treated with dimethyl sulfoxide (DMSO, defined as 0 µM Ros). Treatment with 10 µM Ros led to more decreases of viral titers: 81% reduction in Adwt (P = 0.00001) and 87% reduction in Adhz63 (P = 0.012). The repressed viral yields are consistent with the CPE phenomenon in Figure 5A.

**Figure 6:**
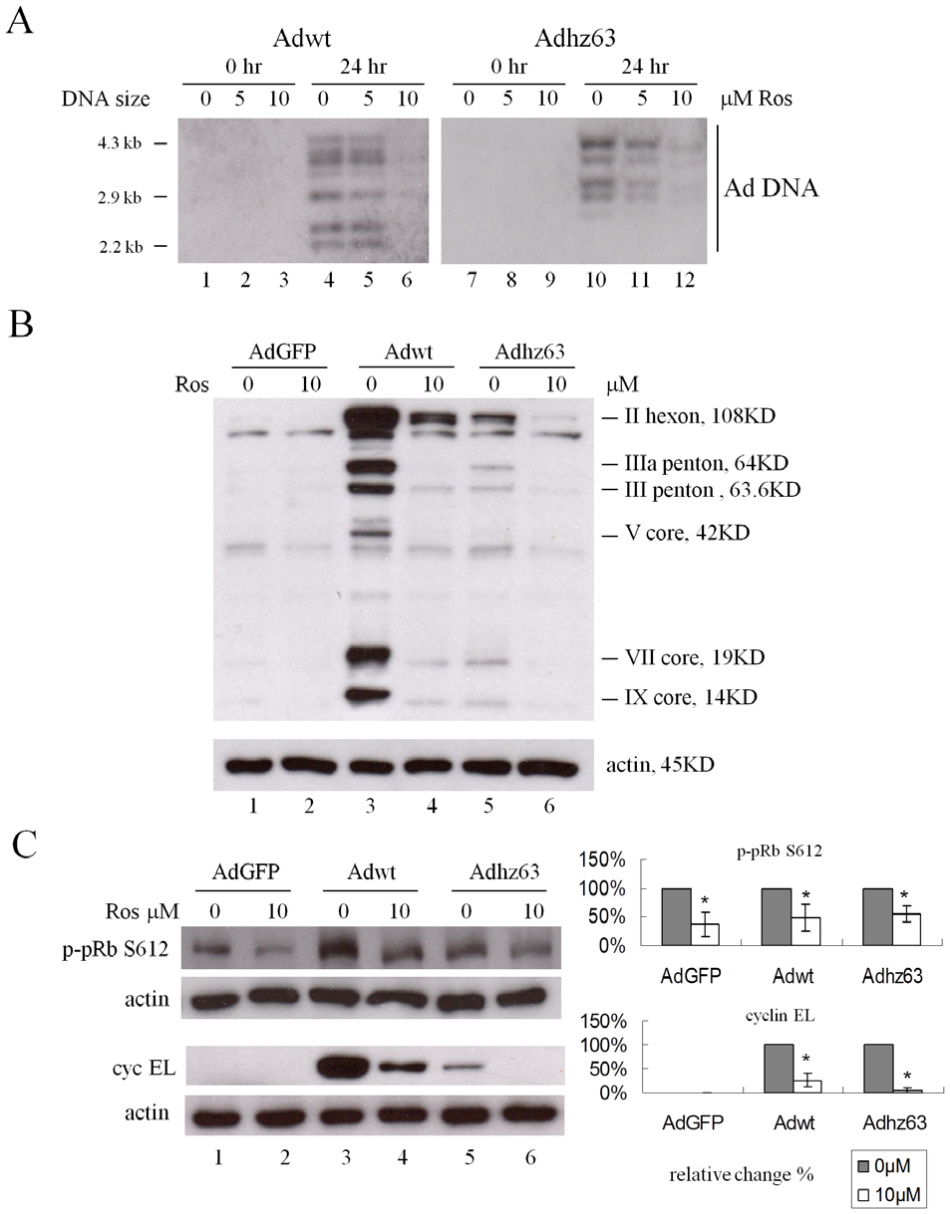
Effects of Ros on viral DNA synthesis, viral capsid proteins, virus-induced cyclin E and phospho-pRb S612. (A) A549 cells were collected at 0 h and 24 h p.i. Viral DNA synthesis was determined by Southern blot. At 24 h p.i., cells were harvested and cell lysates were immunoblotted for (B) adenovirus type 5 capsid proteins, (C) cyclin EL, p-pRb S612, and actin. Actin was used as a loading control. * P<0.05 compared with the 0 µM roscovitine-treated group, Student's t-test.

We then examined the levels of viral DNA and proteins produced in cells affected by Ros treatment. The viral DNA synthesis was determined by Southern blot probed with the Ad genome. The linear Adwt DNA is 36Kb with total 28 *Pst*I restriction sites. The largest fragment is 4333 bp and the smallest fragment is only 12 bp. The sizes of the representative DNA fragments were marked on Figure 6. The viral DNA synthesis of Adwt and Adhz63 at 24 h p.i. was strongly inhibited in the presence of 10 µM Ros (Fig. 6A, lanes 6 and 12). Consistently, the viral capsid proteins were significantly inhibited in the presence of 10 µM Ros (Fig. 6B, lanes 4 and 6). Inhibition of CDK2 activity with Ros reduced the phosphorylation of pRb at the S612 site in AdGFP, Adwt and Adhz63-treated cells (Fig. 6C). Interestingly, Ros treatment markedly repressed the induction of cyclin EL protein caused by Adwt and Adhz63 infection (Fig. 6C, lanes 4 and 6). To sum up, these data show that inhibition of CDK2 with Ros repressed pRb phosphorylation and inhibited viral replication.

### siRNA inhibiting CDK2 represses adenoviral replication by preventing pRb phosphorylation

**Figure 7:**
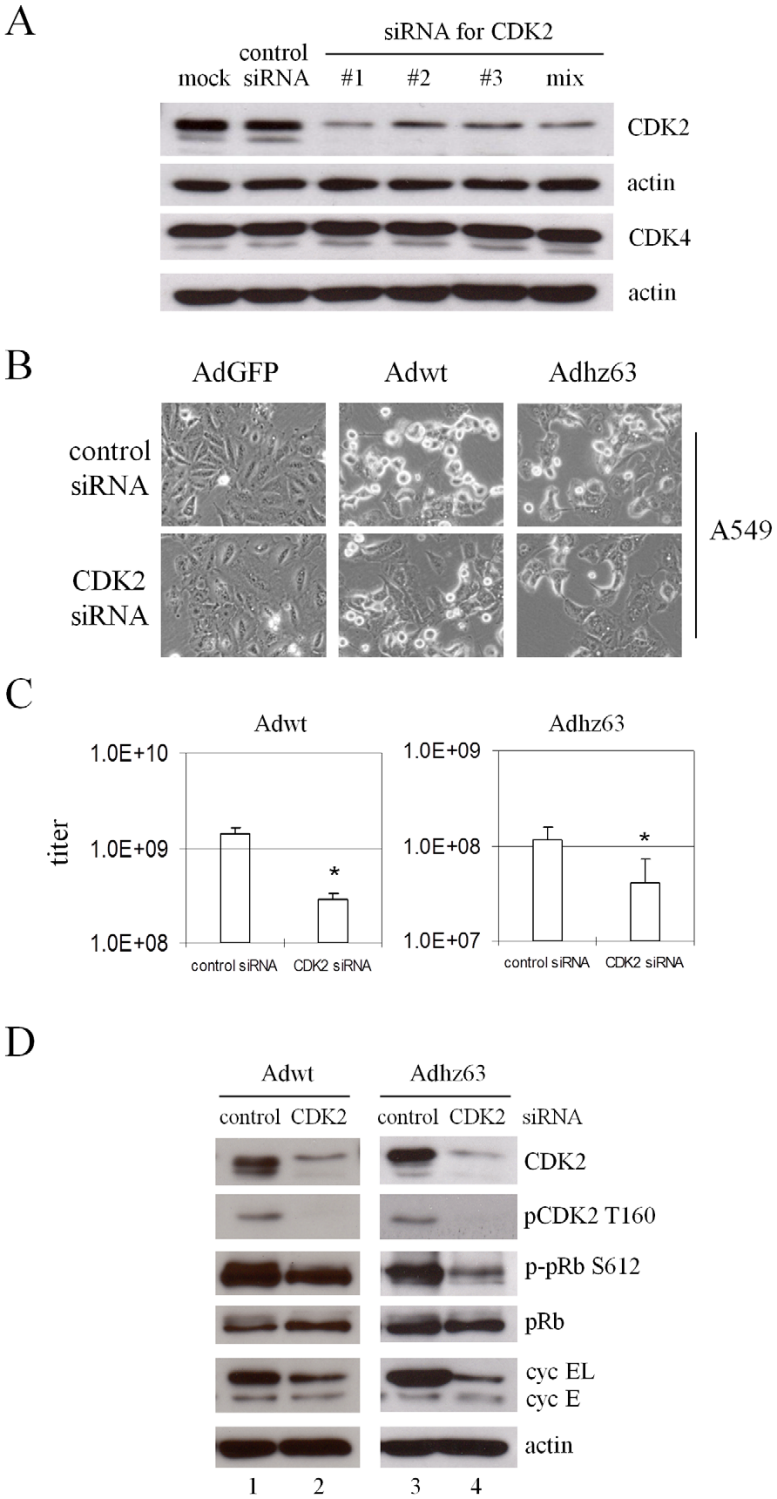
Effects of CDK2-specific siRNA on Ad replication in A549 human lung cancer cells. (A) A549 cells were transfected with 200 nM siRNA duplexes targeting different coding regions of CDK2. “Mix” represents the mixture of three pairs of siRNA duplexes (#1, #2 and #3). Cells were harvested at 48 h after transfection. Cell lysates were immunoblotted for CDK2, CDK4, and actin. (B) At 48 h after transfection with CDK2 siRNA duplex or a duplex of non-specific control siRNA, cells were infected with Adwt or Adhz63 at an MOI of 5. CPE was photographed at 48 h p.i. All microscopy is originally at a magnification of x100. (C) The viral titers were determined at 48 h p.i. with the infection unit method. The values are means ± S.D. of independent triplicate. * P<0.05 compared with the control group, Student's *t*-test. (D) The infected cells were harvested at 24 h after infection. Cell lysates were immunoblotted for CDK2, pCDK2 T160, pRb, p-pRb S612, cyclin E, cyclin EL, and actin. Actin was used as a loading control.

Since the chemical inhibitor Ros may also influence other CDK and kinases [79], we also applied RNA interference to specifically silence CDK2 expression. We tested three different pairs of siRNA duplexes targeting CDK2 on the coding region and showed that all CDK2 siRNAs dramatically inhibited CDK2 expression without the detectable influence on the non-targeted CDK4 in A549 cells (Fig. 7A). To evaluate the effects of CDK2 on the cellular protein production in response to viral infection, A549 cells were infected with Adwt or Adhz63 after treatment with CDK2 siRNA duplex or a non-specific control siRNA for 48 hours. Figure 7B, representing one of the three repeated experiments, shows that blockage of CDK2 expression with siRNA partially inhibited Adwt and Adhz63-induced CPE. Inhibition of CDK2 expression with siRNA resulted in the decreases of Adwt titer from 1.4×10^9^ (control siRNA) to 2.8×10^8^ (specific siRNA) and Adhz63 titer from 1.2×10^8^ to 4.2×10^7^ (Fig. 7C). The effect of CDK2 siRNA on viral replication is statistically significant; the titers decreased about 5 fold for Adwt (P = 0.0005) and 3 fold for Adhz63 (P = 0.03).

We also evaluated the effects of CDK2 on the cellular protein production in response to viral infection. As we expected, the CDK2 siRNA specifically repressed the production of CDK2 protein and decreased pCDK2 T160 in Adwt and Adhz63-infected cells (Fig. 7D, lanes 2 and 4). Repression of CDK2 also resulted in reduced CDK2-specific phosphorylation on pRb, but did not decrease pRb protein levels. In addition, we observed that treatment with CDK2 siRNA repressed specifically Ad-induced cyclin EL, but not cyclin E (Fig. 7D, lanes 2 and 4). This agreed our findings with Ros (Fig. 6C) showing that specifically inhibiting CDK2 with siRNA significantly repressed viral production, which correlated with the decreased CDK2 activation and phosphorylation on pRb in cancer cells.

### siRNA inhibiting CDK2 repressed wild-type adenovirus replication in normal cells

**Figure 8:**
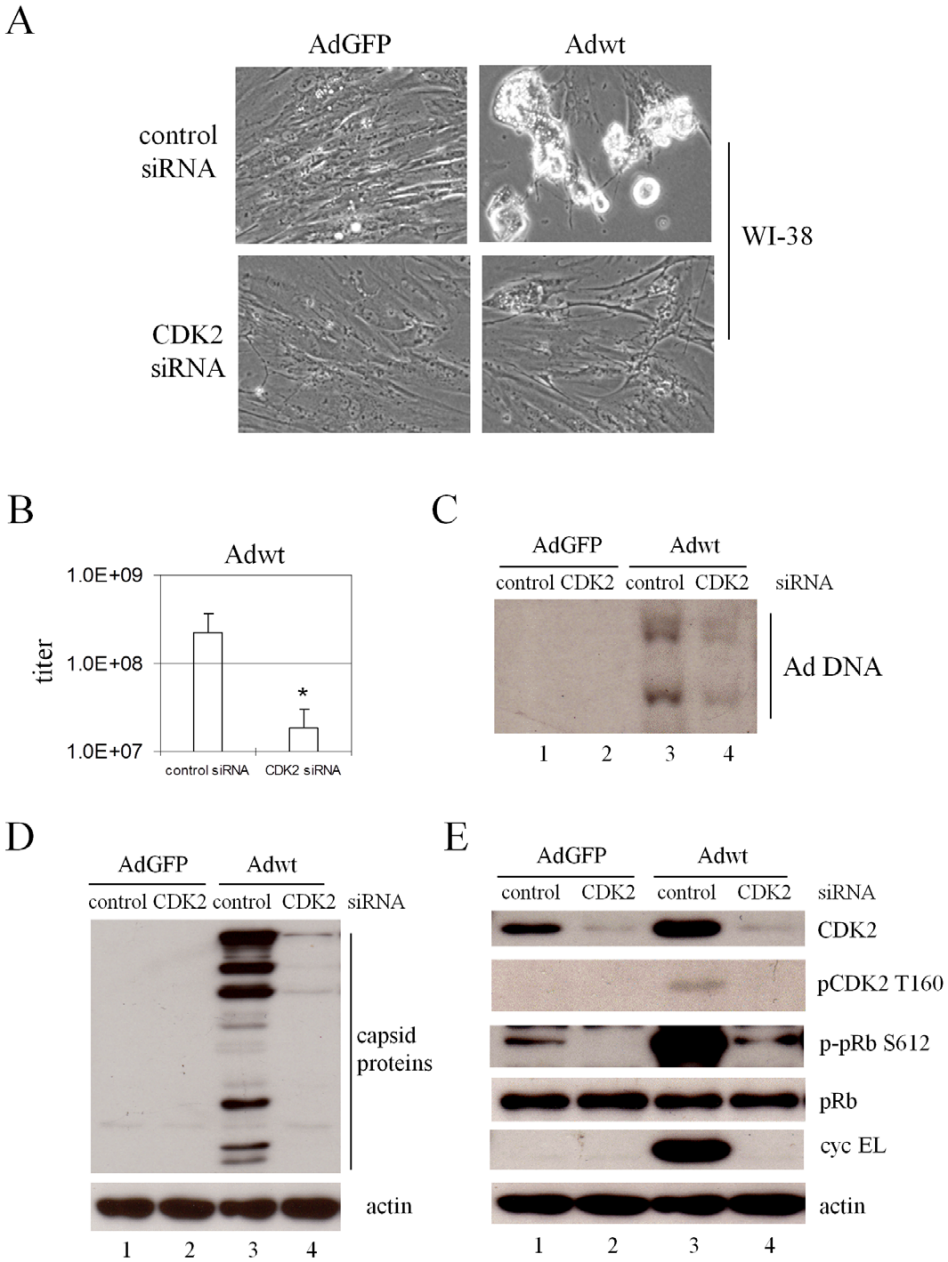
Effects of CDK2-specific siRNA on Ad replication in WI-38 human lung fibroblast cells. WI-38 cells were transfected with CDK2 siRNA duplex or a duplex of non-specific control siRNA. At 48 h after transfection cells were infected with AdGFP, Adwt, or Adhz63 at an MOI of 5. (A) CPE was photographed at 72 h p.i. All microscopy was originally at a magnification of x100. (B) The viral titers were determined at 72 h p.i. with the infection unit method. The values are means ± S.D. of independent triplicate. * P<0.05 compared with the control group, Student's *t*-test. (C) Cells were collected at 48 h p.i. and viral DNA synthesis was determined by Southern blot. Cell lysates were immunoblotted (D) for adenovirus type 5 capsid proteins and for (E) CDK2, pCDK2 T160, pRb, p-pRb S612 and cyclin EL. Actin was used as a loading control.

Considering that the control of G_1_ exit is generally abnormal in cancer cells, we verified the role of CDK2 in Ad replication in WI-38 human diploid cell line that was derived from the normal embryonic lung tissue [80]. As Adhz63 poorly induces cyclin EL and replicates in WI-38 cells (Fig. 1), we therefore investigated Adwt replication in WI-38 cells with the non-replication AdGFP as a negative control. WI-38 cells were infected with AdGFP or Adwt after transfected with CDK2 siRNA duplex or a non-specific control siRNA for 48 h. Figure 8A, representing one of the three repeated experiments at 72 h p.i., shows that blockage of CDK2 expression with siRNA partially inhibited Adwt-induced CPE as we observed with Adwt-infected A549 cells. Inhibition of CDK2 expression with the specific siRNA caused a significant decrease of Adwt titer from 2.2×10^8^ to 1.8×10^7^ (P = 0.03, Fig. 8B). In addition, CDK2 repression also dramatically inhibited the viral DNA synthesis (Fig. 8C, lane 4) and capsid protein production (Fig. 8D, lane 4).

The level of cellular proteins in response to viral infection altered by CDK2 inhibition was also examined in WI-38 cells. For cells treated with the non-specific control siRNA, we were unable to detect pCDK2 T160 and cyclin E in WI-38 cells treated with AdGFP (Fig. 8E); this is related to the strict control of cyclin E expression in WI-38 cells. Adwt infection significantly increased CDK2, pCDK2 T160, and cyclin EL (Fig. 8E, comparing lanes 1 and 3). Inhibition of CDK2 by the siRNA repressed the pCDK2 T160, phospho-pRb S612, and cyclin EL induced by Adwt infection (Fig. 8E, comparing lane 1 with 2 and lane 3 with 4). Adwt infection and siRNA treatment did not show significant effects on pRb (Fig. 8E). Taken together, the results suggest that CDK2 activated by Ad-induced cyclin EL plays a general and important role in the adenoviral replication in normal cells.

## Discussion

**Figure 9:**
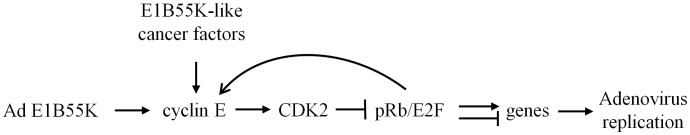
Proposed mechanism of cyclin E function in Ad replication. In Ad-infected cells, Ad E1B55K has a function to enhance cyclin E expression. This E1B55K function is not required for virus replication in cancer cells, which may provide E1B55K-like factors to relax cyclin E regulation and promote cell cycle progression. Cyclin E binds to and activates CDK2. Subsequently, the active pCDK2 phosphorylates the transcriptional repressor pRb, leading to controlling expression of multiple genes, including cyclin E, to provide a suitable cellular environment for viral replication.

By using multiple cell lines (A549, WI-38, HCT116, RKO, HepG2, Hep3B, Saos2, HeLa, MDA-MB-231, and HT29), we previously have shown that induction of cyclin EL is required for Ad replication and correlated with oncolytic selectivity of *E1B55K*-deleted Ad [33], [34]. In this report, we extended the study and focused on cyclin E and CDK2 interaction in human lung cells that are natural host cells for human adenoviruses; we demonstrated that CDK2 activation by cyclin EL is a critical molecular step in Ad replication. Three lines of evidence support the importance of activation of CDK2 by cyclin EL in Ad replication. First, Ad-induced cyclin EL directly interacted with CDK2 and formed cyclin EL/CDK2 complex, leading to specifically increased phosphorylation of CDK2 and pRb (CDK2 at T160 and pRb at S612). Second, the CDK2 chemical inhibitor roscovitine decreased viral replication. Finally, the siRNA specifically inhibiting CDK2 repressed the viral replication with the decrease in pRb phosphorylation. These three lines of evidence support the hypothesis that Ad-induced cyclin EL activates CDK2, which targets the transcriptional suppressor pRb, controlling cellular and viral gene expression for productive viral replication (Fig. 9).

Cyclin E and the large form cyclin EL are generated from alternative splicing. The translation of cyclin EL is initiated at an ATG codon located in exon 2 and cyclin E is from the ATG codon in exon 3 [35]. It has been reported that cyclin EL is found predominantly in breast tumor cells with the abundant lower-molecular-weight (LMW) isoforms [81], [82]. We previously constructed a plasmid, pTet-cycE, containing cyclin E cDNA that produces these two forms of cyclin E proteins [34]. With this approach we clarified that the A549 cell line constitutively expresses the regular cyclin E protein (cyclin E), and Ad infection mainly induces the expression of large form cyclin E protein (cyclin EL) [34]. It is still unclear why Ad infection mainly induces cyclin EL. Considering that cyclin E has CDK2-dependent [53] and independent functions that are related to participation in DNA replication licensing [54] and oncogenic transformation [55], we studied whether Ad-induced cyclin E may target and activate CDK2 in virus-infected cells for productive Ad replication.

We first examined the physical interaction between virus-induced cyclin EL and CDK2. The results indicated that Ad-induced cyclin EL preferentially associates with CDK2 protein (Fig. 2). We cannot exclude that endogenous cyclin E in A549 cells may also interact with CDK2; however, we observed that the increased CDK2 in the cyclin E/CDK2 immunocomplexes was associated with cyclin EL induction after infection with replication-competent Adwt and Adhz63. The results suggested cyclin EL highly interacts with CDK2 in Ad-infected cancer cells. In proliferating cells, the abundance of the cyclin E protein directly links to the formation of active cyclin E/CDK2 complex [35], [83]. With an intact cyclin box and the C-terminal 50 amino acids, the full-length cyclin EL is able to bind and activate CDK2 as cyclin E [53], [55]. Consistent with our finding, previous studies showed that the addition of exogenous cyclin EL increases the formation of cyclin EL/CDK2 complex correlating to the increased activity and phosphorylation of CDK2 in human lung fibroblasts [35] and breast cancer cells [84]. We reason that Ad-induced cyclin EL may have a strong affinity to CDK2 in the cellular environment affected by Ad infection.

We identified that Ad-induced cyclin EL correlates with the increase in phosphorylation of CDK2 at T160 and pRb at S612 (Figs. 3B and 4A). Three phosphorylation sites have been identified in CDK2 [66]. T160 phosphorylation is essential for CDK2 activity, while T14 and Y15 phosphorylation cause an inhibitory effect. The retinoblastoma tumor suppressor pRb is inactivated by CDK's phosphorylation and enables E2F transcription factor to be released from the pRb/E2F complex to carry out the downstream gene regulation [85], [86]. Phosphopeptide analysis of pRb showed that S612 is one of the CDK2-preferred phosphorylation sites [71]. We also examined the level of pRb with phosphorylation of T821 (CDK2-preferred) and S795 (CDK4-preferred); we did not detect any significant change at either of these two sites (Fig. 4A). Inhibition of CDK2 expression with the CDK2 siRNA repressed phosphorylation on CDK2 and pRb (Fig. 7D) and decreased viral replication (Fig. 7C). These results indicate that Ad-induced cyclin EL activates CDK2 by phosphorylating at T160, which then specifically introduces pRb phosphorylation at the S612 site.

The pRb phosphorylation by cyclin EL/CDK2 may lead to regulation of multiple cellular and viral genes for productive Ad replication. Interestingly, Ad-induced cyclin EL expression was also inhibited by a CDK2 chemical inhibitor and CDK2 siRNA (Figs. 6C, 7D and 8E). It seems that inhibition of CDK2 interferes in the cyclin E induction via a loopback regulation (Fig. 9). Previous studies have reported that cyclin E gene is the downstream target of E2F [87], [88]. In our previous work, we showed that the cyclin E promoter is more active in cancer cells and the promoter activity is further enhanced after Ad infection [34]. We suggest that cyclin EL activates the cyclin EL-CDK2-pRb/E2F pathway and cyclin EL itself is also one of the targets of the pathway.

We detected a notable decrease of CDK inhibitors p21 and p27 in the Ad-infected cells. p21 and p27 inhibit the activity of cyclin/CDK complexes to prevent the cell-cycle progression, and their protein stability is also regulated by cyclin/CDK complexes [75], [89], [90]. Phosphorylation of p27 by cyclin E/CDK2 causes p27 degradation [89], [91]. Montagnoli *et al.* (1999) showed that cyclin E/CDK2-dependent phosphorylation of p27 at threonine 187 facilitates the formation of a trimeric complex with cyclin E/CDK2 and leads to p27 ubiquitination [92]. In agreement with our findings, recent studies also suggest that CDK may promote p21 degradation [90], [93]. Thus, the activated cyclin E and CDK2 may decrease the CDK inhibitors p21 and p27 to benefit viral replication.

In summary, our results showed that Ad-induced cyclin EL binds to and activates CDK2 that subsequently phosphorylates the transcriptional suppressor pRb, which can regulate expression of multiple cellular and viral genes, including cyclin E (Fig. 9). Our previous studies have shown that Ad E1B55K has a function to enhance cyclin E induction. In cancer cells, this E1B55K function is not critically required for cyclin E induction and viral replication, likely because of deregulated cyclin E expression or having E1B-like cancer cellular factors. This study demonstrated that Ad-induced cyclin EL plays a critical role in Ad replication through activation of CDK2 that generates a suitable environment for viral replication. Our study reveals a new molecular basis for Ad replication in cancer cells that will guard us to develop new oncolytic vectors and therapeutic strategies.
